# Ocean and High Altitude Health Resorts

**Published:** 1899-11

**Authors:** 


### Ocean and High Altitude Health Resorts.

Dr. A. N. Bell (*The Sanitarian*) holds that recent knowledge of microbic life justifies the inference that the benefit to consumptives derived from sea voyages or high altitudes is independent alike of the extreme density and moisture of the air in one, or of rarefaction and dryness in the other. Both are inimical to microbic life, and are alike propitious and commendable to persons afflicted with or predisposed to pulmonary consumption. The special advantages of an ocean atmosphere are its entire freedom from the dust common to domestic conditions and complete change of scene and rest, together with relief from all sources of excitement and worry.—*Indian Lancet.*

